# Vesicular Disease Caused by Seneca Valley Virus in Pigs, England, 2022

**DOI:** 10.3201/eid3202.251194

**Published:** 2026-02

**Authors:** Bryony Armson, Valérie Mioulet, Britta A. Wood, Antonello Di Nardo, Nick J. Knowles, Jemma Wadsworth, David J. Paton, Jozhel Baguisi, Harry Bull, Amy McCarron, Clare Browning, Ashley Gray, Tomasz Zaleski, Andrew E. Shaw, Anna B. Ludi, Mark Henstock, Hayley M. Hicks, Ginette Wilsden, Krupali Parekh, Julie Maryan, Sarah Belgrave, Noemi Polo, Simon Gubbins, Claire Colenutt, Melanie Nicholls, Emma Brown, Efthymia Nasou, Anca Drelciuc, Livio Pittalis, David Jorge, Caroline Wilson, Susana Taylor, Jose Bis, Charles Nfon, Susanna Williamson, Donald P. King

**Affiliations:** The Pirbright Institute, Woking, UK (B. Armson, V. Mioulet, B.A. Wood, A. Di Nardo, N.J. Knowles, J. Wadsworth, D.J. Paton, J. Baguisi, H. Bull, A. McCarron, C. Browning, A. Gray, T. Zaleski, A.E. Shaw, A.B. Ludi, M. Henstock, H.M. Hicks, G. Wilsden, K. Parekh, J. Maryan, S. Belgrave, N. Polo, S. Gubbins, C. Colenutt, M. Nicholls, E. Brown, D.P. King); University of Cambridge, Cambridge, UK (T. Zaleski); Animal and Plant Health Agency, Bury St Edmunds, UK (T. Zaleski, E. Nasou, A. Drelciuc, L. Pittalis, D. Jorge, C. Wilson, S. Taylor, J. Bis, S. Williamson); National Centre for Foreign Animal Disease, Canadian Food Inspection Agency, Winnipeg, Manitoba, Canada (C. Nfon)

**Keywords:** Vesicular disease, Seneca Valley virus, viruses, *Senecavirus valles*, pigs, England

## Abstract

Vesicular disease caused by Seneca Valley virus infection occurred in pigs from 5 outdoor pig farms in England during June–September 2022. Clinical signs resembled notifiable vesicular diseases, such as foot-and-mouth disease. Full genome sequences shared a common ancestor with a virus circulating in the United States.

Researchers reported vesicular disease associated with Seneca Valley virus (SVV; *Senecavirus valles*, family Picornaviridae) in pigs imported into the United States from Canada in 2007 (*1*). Similar reports subsequently emerged from other countries, including Brazil, China, Thailand, Chile, India, Vietnam, Columbia, and Mexico (*2*–*4*). We describe cases of SVV infection in pigs from 5 pig breeding farms in eastern England during June–September 2022. 

Farm staff initially observed signs of vesicular disease in recently inseminated sows at an outdoor breeding unit (SVV2022-01): lameness, reluctance to move, and lesions on the nose and feet, varying from discrete vesicles on the coronary band and interdigital space to deep erosions and heel horn separation. We collected blood and vesicular tissue samples as part of an official vesicular disease investigation; all samples tested negative by real-time reverse transcription PCR (rRT-PCR) for notifiable diseases (foot-and-mouth disease virus, swine vesicular disease virus, and vesicular stomatitis virus) (*5*). However, we observed cytopathic effect during virus isolation, and parallel rRT-PCR testing (*6*) generated positive results for SVV.

We subsequently identified vesicular disease in recently inseminated sows on 3 additional farms (SVV2022-02 [Figure 1], SVV2022-03 and SVV2022-05). Again, official veterinary investigations yielded negative results for notifiable diseases and confirmed the presence of SVV by rRT-PCR. Gilts, young boars, and weaners appeared clinically unaffected, despite evidence of SVV in rectal and nasal swab specimens. Retrospective tracing identified another farm (SVV2022-04) with confirmed SVV in a group of recently lame sows.

**Figure 1:**
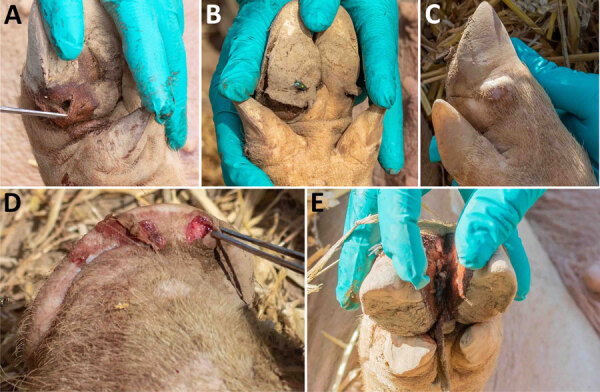
Affected pigs on farm SVV2022-02, from study of vesicular disease caused by Seneca Valley virus in pigs, England, 2022. Vesicular lesions can be seen on the coronary bands (A-C), snout (D), and interdigital cleft (E). Hoof horn separation also occurred in some infected pigs (B). Some lesions resembled those of foot-and-mouth disease (D), but others were more deep-seated (A).

We collected samples including vesicular epithelium, vesicular fluid, rectal and nasal swabs, blood, and tonsils from dead pigs. We also collected samples from weaners derived from 4 of the 5 affected farms and from sows and postmortem pigs at farm SVV2022-03 for up to 4 months after the initial disease reports. In total, 461 (35.0%) of 1,319 samples tested positive for SVV by rRT-PCR from the 5 farms (Appendix Table). On farms SVV2022-01 and SVV2022-02, we initially collected blood samples, with 17 of 34 positive by rRT-PCR; however, because viremia is short-lived, that sampling matrix was not ideal for surveillance. Analysis revealed the highest viral loads in vesicular lesion and tonsil samples (strongest cycle threshold value 10.8). Rectal swabs were the most frequently collected sample type (n = 914) owing to ease of collection. Nasal swab specimens were useful in revealing acute stages of disease, but rectal swab specimens proved more useful in detecting SVV in recovering pigs, despite weaker rRT-PCR responses. That observation supports the use of rectal swab sampling in pigs of unknown SVV status, where resources or logistics limit sampling options. Our data also highlight the value of testing tonsils, illustrated by detection of SVV RNA in a tonsil from a dead piglet >35 days after the episode of clinical signs (farm SVV2022-02).

We conducted serologic investigations 5 weeks after the disease episode at farm SVV2022-04 and during the acute stage of disease at farm SVV2022-01. A total of 55 of 63 serum samples from farm SVV2022-01 and 10 of 10 samples from farm SVV2022-04 were positive for SVV-specific antibodies as determined by the virus neutralization test using SK6 cells.

Paired rectal and semen samples collected from boars supplying semen and historic batches of feed and soya bean meal samples supplied to affected farms all tested negative for SVV by rRT-PCR. We detected SVV RNA in 76 (56.7%) of 134 environmental samples (*7*) collected 3.5 weeks after the disease occurrence from farm SVV2022-01, where pigs no longer remained on the premises. Sample sites included walls, doors, feeders, drinkers, floors, gates, and a trailer. We also detected SVV RNA in 6 (10.2%) of 59 samples collected 6 weeks after the disease occurrence from farm SVV2022-04, where pigs remained (sites included loading area, drinker, ark, and trailer) (Appendix Figure). Our data highlight the importance of cleaning, disinfection, and stringent biosecurity to limit the spread of SVV.

We characterized SVV isolates using next-generation sequencing (*8*) and found they share a common ancestor with a virus isolated in the United States during 2020 (SVV/USA/TN/NADC6/2020; GenBank accession no. MZ733975) (Figure 2), predicted to have circulated around November 2020 (95% highest posterior density June 2020–March 2021). The SVV sequences were assigned into 2 sister clades differing at >50 nt sites, consistent with 2 possible epidemiologic scenarios: a single virus introduction, with the resulting diversity accruing from within-country transmissions and evolution; or independent introductions into England of viruses characterized by a slightly different genetic signature. Further epidemiologic investigation could determine the most important risk pathways for introduction, transmission routes between farms, and geographic spread of SVV infection in the United Kingdom.

**Figure 2:**
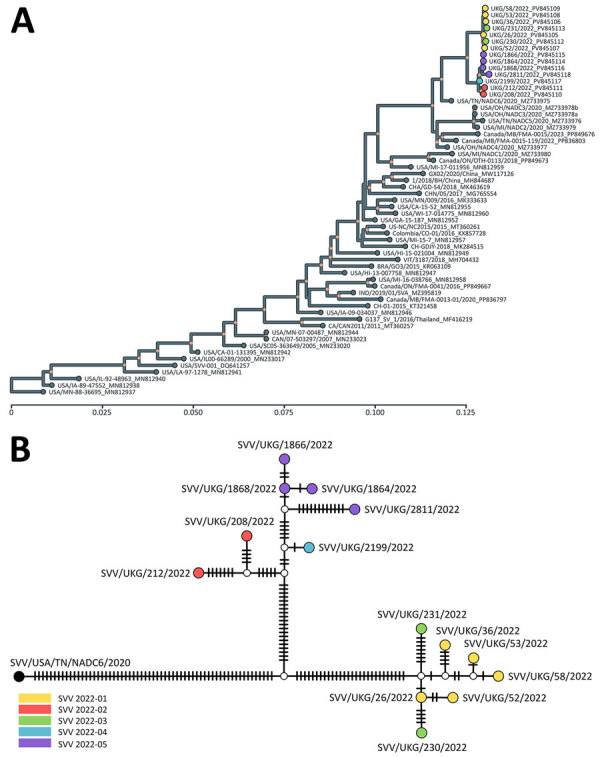
Evolutionary history and genetic relationships of Seneca Valley viruses from study of vesicular disease caused by Seneca Valley virus in pigs, England, 2022. A) Tree represents the evolutionary history of Seneca Valley viruses isolated globally and reconstructed using polyprotein-coding sequences. Maximum-likelihood tree inferred using the Tamura-Nei model (*9*) and setting a discrete gamma distribution for evolutionary rate differences among sites. Colored tips represent Seneca Valley virus–infected farms during the outbreak in England in 2022. Colored internal nodes represent the percentage of trees in which the associated taxa clustered together on >50%. Evolutionary analyses were conducted in MEGA11 (*10*). Scale bar indicates nucleotide substitutions per site. B) Genetic relationship of Seneca Valley viruses isolated in England during 2022 based on the full-genome length, as reconstructed by statistical parsimony analysis. Nodes are colored according to farm on which clinical cases were observed; white nodes denote missing unsampled haplotypes. Hatch marks represent single-nucleotide substitutions estimated between the connected nodes.

In conclusion, the clinical similarity of the SVV disease outbreaks we describe to notifiable vesicular diseases highlights the value of passive surveillance and the legal requirement for pig keepers and veterinarians to report vesicular lesions promptly. Cases of SVV infection were transient, and pigs recovered quickly, with minimal productivity losses. We alerted regional veterinarians and farmers of the need to remain vigilant for vesicular disease, and there have been no further clinical cases of SVV in England since September 2022. 

Appendix Additional information for vesicular disease caused by Seneca Valley virus in pigs, England, 2022.
