# Supplementary material for: Vesicular Disease Caused by Seneca Valley Virus in Pigs, England, 2022
Source: Emerg Infect Dis. 2026 Feb;32(2):295–9. doi: 10.3201/eid3202.251194 (PMC12928217; doi:10.3201/eid3202.251194)
Supplement: Appendix — Additional information for vesicular disease caused by Seneca Valley virus in pigs, England, 2022. [file 25-1194-Techapp-s1.pdf]

Article DOI: http://doi.org/10.3201/eid3202.251194

EID cannot ensure accessibility for supplementary materials supplied by authors. Readers who have difficulty accessing supplementary content should contact the authors for assistance.

Vesicular Disease Caused by Seneca Valley Virus in Pigs, England, 2022

Appendix

Appendix Table. Five affected farms studied in an investigation of vesicular disease caused by SVV in pigs, England, 2022, with details of clinical samples tested by SVV real-time reverse-transcription PCR assay*

	Date of first	SVV rRT-PCR results, positive samples/total samples tested							
	sample		Vesicular		Rectal	Nasal	Other		
Farm	collection	All samples	lesion†	Tonsil‡	swab	swab	swab§	Blood¶	Other#
SVV 2022–01	23/06/2022	102/174	2/2	11/13	41/64	34/61	7/10	7/24	0/0
SVV 2022–02	12/07/2022	148/436	16/16	11/14	72/315	40/81	0/0	9/10	0/0
SVV 2022–03	14/07/2022	147/350	21/21	0/12	93/235	0/0	33/45	0/0	0/37
SVV 2022–04	21/07/2022	11/106	0/0	3/3	8/103	0/0	0/0	0/0	0/0
SVV 2022–05	02/09/2022	53/253	15/15	3/10	20/197	15/15	0/0	0/0	0/16
Total		461/1319	54/54	28/52	234/914	89/157	40/55	17/34	0/53

*SVV, Seneca Valley virus; rRT-PCR, real-time reverse-transcription PCR
†Includes vesicular fluid and swabs
‡Includes tonsil swab
§includes throat swab and fecal swab
¶includes serum and EDTA blood
#Other includes tissue from the forelimb skin, tongue, coronary band, snout, nasal turbinate, spleen or liver, where vesicular lesions were absent.

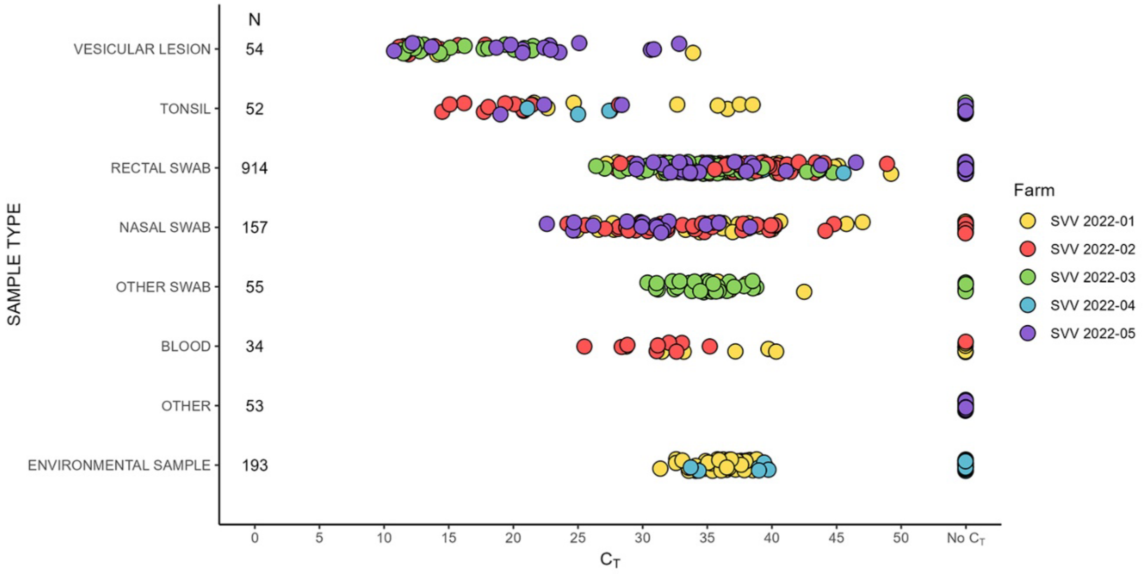

Appendix Figure. Cycle threshold values of samples tested by Seneca Valley virus real-time reverse transcription PCR. Sample descriptions are the same as for Appendix Table. N = number of samples tested for each sample type. “Other” includes tissue from the forelimb skin, tongue, coronary band, snout, nasal turbinate, spleen or liver, where vesicular lesions were absent.
